# A rare variation of duplicated portal vein: left branch derived from splenic vein mimicking cavernous transformation

**DOI:** 10.1186/s12876-021-01970-8

**Published:** 2021-10-26

**Authors:** Qian Yang, Jun Li, Hanwei Wang, Shunan Wang

**Affiliations:** 1 Department of Radiology, Daping Hospital, Army Medical University, Chongqing, 400042 China; 2 Clinical Research Center of Imaging and Nuclear Medicine, Chongqing, 400042 China

**Keywords:** Anatomic variant, Duplication of the portal vein, Computed tomography, Case report

## Abstract

**Background:**

Duplication of the portal vein is a rare type of anatomic variant of the portal vein (PV) system that can be incidentally found and can lead to various challenges and consequences. Herein, we report an unusual case to increase our understanding of such anatomic variants.

**Case presentation:**

A 67-year-old asymptomatic woman was diagnosed with a liver space-occupying lesion by ultrasonography on a routine physical examination. The laboratory examinations from a local hospital suggested that her liver function tests were normal. The liver appeared normal on pre-contrast enhanced CT images. However, there were multiple complex abnormalities of PV found on contrast-enhanced CT scans, including two independent sources of PV (duplication), preduodenal PV, circum-portal pancreas, mimic cavernous transformation, abnormal branches of PV, and transient abnormal perfusion in the left lobe of the liver. MRI showed fatty infiltration in the left lobe of the liver.

**Conclusion:**

This case extends our current understanding of the anatomical variations of the PV system. Knowledge of these complex and rare anatomical variations will help clinical doctors make a confident diagnosis or assist with proper planning of a surgical procedure.

## Background

The development and formation of the portal venous system is a complex process. Anatomic variants and congenital hypoplasia of the portal system can be found in 20–35% of individuals. Duplication of the portal vein is a rare type of anatomic variant of the portal vein (PV) system [1, 9]. With the development of medical imaging equipment and the improvement of inspection technology, duplication of the portal vein is discovered more often than before and lead to some challenges and consequences, especially for junior residents. Herein, we report an unusual case of duplication with other complex abnormalities of the PV. This case is not consistent with the previously reported common anatomic variant or acquired abnormality classification of the PV.

## Case presentation

A 67-year-old asymptomatic woman was hospitalized, because she was suspected of having a liver space-occupying lesion diagnosed by ultrasonography on a routine physical examination. She was referred to our hospital for further assessment of the liver mass by computed tomography (CT) and magnetic resonance imaging (MRI). Laboratory examinations from a local hospital suggested that her liver function tests were normal, and all of her viral hepatitis surface antigens were negative. The alpha foetal protein (AFP) level was 1.5 ng/ml (normal level < 10 ng/ml). She denied any chronic diseases, drug administration, alcohol consumption or venereal exposure.

**Fig. 1:**
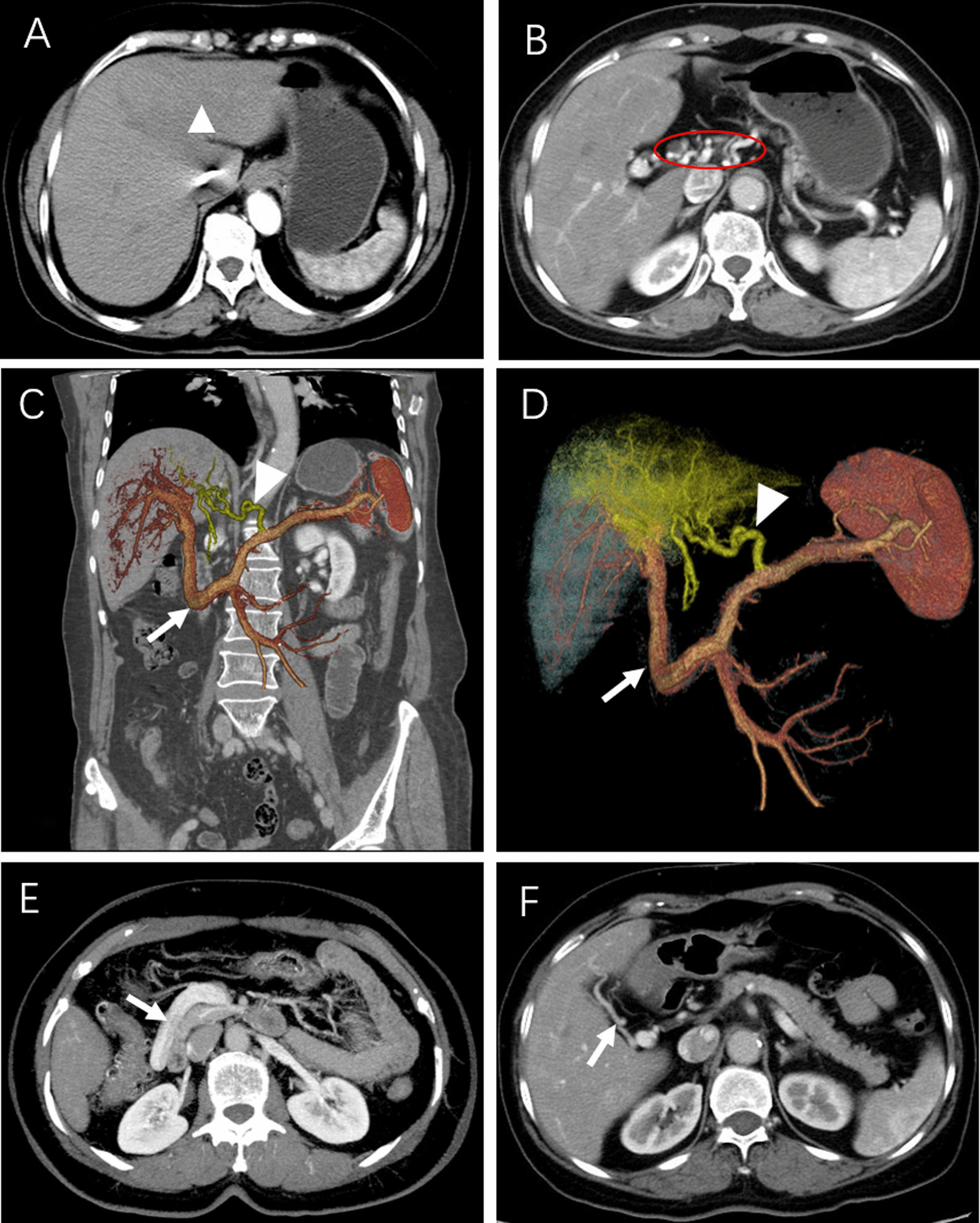
Post-contrast enhanced CT scans of the patient’s abdomen. **a** Transient perfusion abnormal region at the left lobe of liver (arrowhead). **b** Tortuous vessels mimic cavernous transformation of the PV (red circle). **c–e** The VR and MIP images showed SMV and SV join to form a preduodenal portal vein (PV-1) (arrow), and another portal vein (PV-2) (arrowhead) derives from the SV. **f** The PV-1 directly divided one branch supplied segment IV (arrow)

**Fig. 2:**
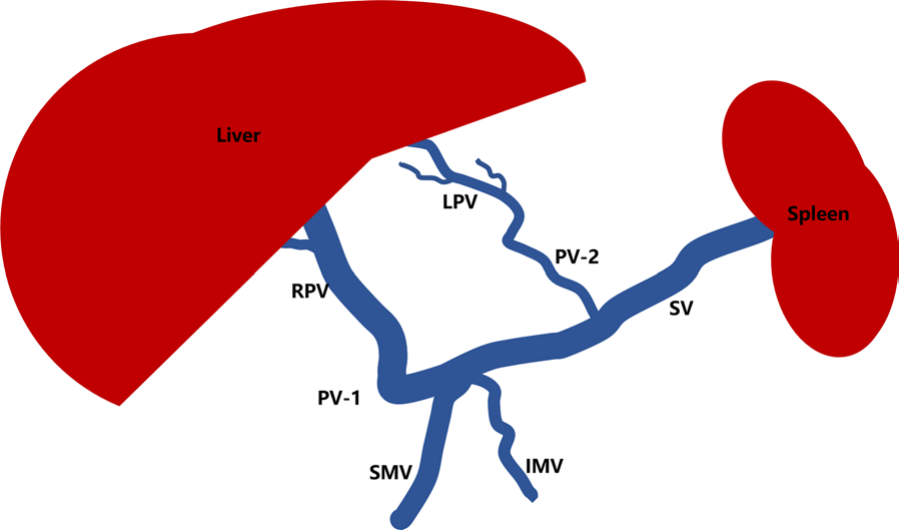
A schematic diagram showing the duplicated portal vein anomaly in our case

**Fig. 3:**
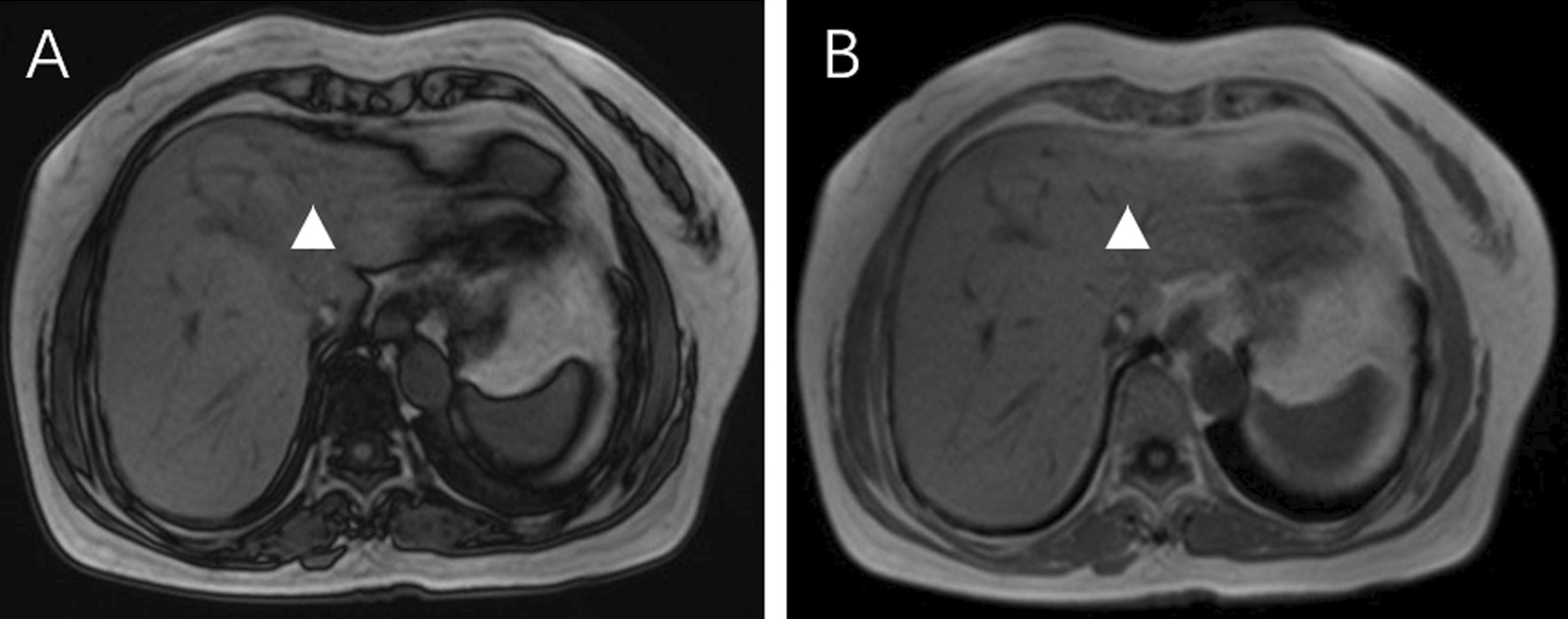
Patient showed fat accumulation in left lobe of liver. **a** T1 out-phase image showed the hypointensity of left liver lobe(arrowhead). **b** T1 in-phase image showed the homogeneous signal of left liver lobe (arrowhead)

Pre-contrast enhanced CT scans of the abdomen showed that the shape, size and density of the liver were normal. On the arterial phase, contrast-enhanced CT images revealed transient perfusion abnormalities in the left lobe of the liver and homogeneous enhancement in both the portal vein phase and delayed phase (Fig. 1a). However, it is worth noting that the CT portal-phase images showed multiple slender and tortuous vessels in the hepatic hilar region, which mimicked cavernous transformation of the portal vein (Fig. 1b). We performed maximum intensity projection (MIP) and volume rendering (VR) postprocessing techniques to review the hepatic vascular system and found that this patient had multiple anatomic variations of the PV system. The portal system consisted of two main branches. PV-1 was formed by the junction of the superior mesenteric vein (SMV) and the splenic vein (SV), anterior to the descending duodenal and pancreatic neck with no thickening or dilatation. PV-1 then divided into two branches: one branch directly supplied segment IV of the liver, and the other branch extended into the right portal vein (RPV) (Fig. 1c, d). The RPV subdivided into the right anterior portal vein (RAPV) and the right posterior portal vein (RPPV). PV-2 was independently derived from the SV and extended a slender/tortuous vessel into the left portal vein (LPV) to supply segments II and III of the liver. There was communication between the RPV and LPV. In order to better know our case, we provided a schematic diagram showing the anatomical variation of the duplicated portal vein (Fig. 2).The hepatic arterial/venous system and inferior vena cava were normal. No abnormal shunt or varix was seen in the abdomen. The in-phase and out-phase T1-weighted imaging on MRI clearly showed fat accumulation in the left lobe of the liver (Fig. 3a, b).

## Discussion and conclusions

Duplication of the portal vein is a rare type of variant of the PV that is only described by case reports. It is defined as two separated portal veins that course upward to the porta hepatis and divide into segmental branches [2, 3]. Duplication of the portal vein usually causes clinically relevant symptoms due to its unusual topography and haemodynamics. In a case described by Dighe and Vaidya [2], the splenic vein and superior mesenteric vein appeared to enter the liver separately and joined to form a small main portal vein within the liver. Another case report described two portal veins, where one was a continuation of the SV in the usual retro-duodenal path, while the other was a preduodenal portal vein from the confluence of the superior and inferior mesenteric veins [4].

In the present case, the PV system was formed by two separated main veins. PV-1 was formed by the normal development and junction of the SMV and the SV, while PV-2 was independently derived from the SV. Such duplication of PV was consistent with previous reports [2, 5]. However, this case has far more complex malformations in the PV system. PV-1 ran anterior to the descending duodenum, and the pancreatic neck entered the liver without duodenal stenosis or common bile duct stricture. Mostly, preduodenal PV is a congenital anomaly of the main portal vein, which may be found in the setting of duodenal obstruction or incidentally at surgery [6]. An increased incidence of preduodenal PV has also been reported in individuals with polysplenia syndrome, situs anomalies or biliary atresia [7, 8].

PV-2 was independently derived from the SV extending a slender/tortuous vessel mimicking cavernous transformation directly into the left portal vein. However, this patient had no symptoms of portal hypertension. We considered that these events occurred due to further regression of the right umbilical vein, whereas the left umbilical vein continued to connect with both the ductus venosus and the left portal vein at a junction called the umbilicoportal confluence during the embryonic period. It has been proposed that aberrations in this process of involution can result in anatomical variations within the PV [9, 10]. Furthermore, we hypothesize that hepatic fatty infiltration and transient hepatic perfusion disorders occur in the left lobe of the liver due to an alteration of haemodynamics caused by aberrant venous inflow into the liver.

As stated before, such PV malformations may directly cause some diseases of the liver, gastrointestinal or bile pancreatic system. They may also give rise to secondary portal hypertension with the development of oesophagogastric varices and may lead to a source of fatal haemorrhage. Although this patient presented with no symptoms, further follow-up examinations should be performed due to the multiple and complex malformations of the PV system, especially due to the unusual topography (preduodenal PV-1) and abnormal haemodynamics (mimic cavernous transformation of PV-2). For doctors, this case presented a rare and complex anatomical variation of the PV system, which could further the understanding of the variations of the PV system. Knowledge of these variations helps in the proper planning of patient management, especially for interventional surgery and laparoscopic surgery.

## Data Availability

The datasets supporting the conclusions of this article are included in the article.

## Abbreviations

PV: Portal vein
PV-1 and PV-2: Two separated main branch of PV system
RPV: Right portal vein
LPV: Left portal vein
RAPV: Right anterior portal vein
RPPV: Right posterior portal vein
SMV: Superior mesenteric vein
SV: Splenic vein
CT: Computed tomography
MIP: Maximum intensity projection
VR: Volume rendering
MRI: Magnetic resonance imaging
